# Epimorphic regeneration of the mouse digit tip is finite

**DOI:** 10.1186/s13287-022-02741-2

**Published:** 2022-02-07

**Authors:** Connor P. Dolan, Tae-Jung Yang, Katherine Zimmel, Felisha Imholt, Osama Qureshi, Alyssa Falck, Joshua Gregory, Macie Mayes, Kayla Ritchie, Hannah Koester, Benjamin Daniel, Mingquan Yan, Ling Yu, Larry J. Suva, Dana Gaddy, Lindsay A. Dawson, Ken Muneoka, Regina Brunauer

**Affiliations:** 1 Department of Veterinary Physiology and Pharmacology, College of Veterinary Medicine and Biomedical Sciences, Texas A&M University, College Station, TX USA; 2 Present Address: DoD-VA Extremity Trauma and Amputation Center of Excellence, Bethesda, MD USA; 3 Present Address: Department of Surgery, Uniformed Services University of the Health Sciences and Walter Reed National Military Medical Center, Bethesda, MD USA; 4 Department of Veterinary Integrative Biosciences, College of Veterinary Medicine and Biomedical Sciences, Texas A&M University, College Station, TX USA

**Keywords:** Regeneration, Amputation, Stem cells, Positional information

## Abstract

**Background:**

Structural regeneration of amputated appendages by blastema-mediated, epimorphic regeneration is a process whose mechanisms are beginning to be employed for inducing regeneration. While epimorphic regeneration is classically studied in non-amniote vertebrates such as salamanders, mammals also possess a limited ability for epimorphic regeneration, best exemplified by the regeneration of the distal mouse digit tip. A fundamental, but still unresolved question is whether epimorphic regeneration and blastema formation is exhaustible, similar to the finite limits of stem-cell mediated tissue regeneration.

**Methods:**

In this study, distal mouse digits were amputated, allowed to regenerate and then repeatedly amputated. To quantify the extent and patterning of the regenerated digit, the digit bone as the most prominent regenerating element in the mouse digit was followed by in vivo µCT.

**Results:**

Analyses revealed that digit regeneration is indeed progressively attenuated, beginning after the second regeneration cycle, but that the pattern is faithfully restored until the end of the fourth regeneration cycle. Surprisingly, when unamputated digits in the vicinity of repeatedly amputated digits were themselves amputated, these new amputations also exhibited a similarly attenuated regeneration response, suggesting a systemic component to the amputation injury response.

**Conclusions:**

In sum, these data suggest that epimorphic regeneration in mammals is finite and due to the exhaustion of the proliferation and differentiation capacity of the blastema cell source.

**Supplementary Information:**

The online version contains supplementary material available at 10.1186/s13287-022-02741-2.

## Background

Multicellular organisms rely on stem cells for tissue homeostasis and repair throughout life [1, 2]. In mammals, tissue damage is repaired by tissue-specific stem cells that are quiescent under homeostatic conditions and activated upon injury to enter the cell cycle and generate rapidly proliferating and differentiating progeny. This reparative mode of tissue regeneration is limited by the size of the damage that can be repaired, as is apparent in volumetric muscle loss injury and bone critical size defects [3, 4], and it is exhaustible. For example, repeated damage of bronchial epithelium leads to loss of stem cells and consequently impaired regeneration [5]. Similarly, hematopoietic stem cell transplantations can be performed for a limited number of times until the recipient hematopoietic system fails to be repopulated [6], and muscle stem cell pools in Duchenne’s muscular dystrophy are eventually exhausted, which leads to failure of muscle function [7].

In contrast to stem-cell mediated reparative tissue regeneration, epimorphic regeneration is a structural regeneration mode that enables vertebrates to regrow all tissues of an amputated structure in a patterned fashion by de novo morphogenesis. As such, salamanders regenerate a perfect replicate of an amputated limb, and mice and humans regenerate the distal digit tip [8]. Epimorphic regeneration is mediated by a blastema that forms by either dedifferentiation or recruitment of tissue-resident stem cells, or a combination of both, as appears to be the case in the mammalian digit tip [9–11]. A fundamental and unanswered question is whether blastema-mediated regeneration is exhaustible, as with stem cell-mediated reparative tissue regeneration, or whether the blastema allows for indefinite regeneration. Studies of repeated amputation injury in lower vertebrates have given an inconclusive answer thus far. For instance, zebrafish (*Danio rerio*) fin regeneration can be repeated for at least 9 times without any apparent defects [12]. Moreover, in an extensive study spanning a total of 16 years and 18 consecutive lentectomies, Japanese newts (*Cynops pyrrhogaster*) were found to perfectly regenerate lenses, regardless of the number of previous lentectomies and the age of the animal [13]. In contrast, the limbs of eastern newts (*Notophthalmus viridescens*) and axolotls (*Axolotl mexicanum*) fail to regenerate after 3–5 amputation–regeneration cycles [14, 15]. Thus, it is impossible to generalize about the limitations of epimorphic regeneration.

The ability to undergo epimorphic regeneration is not restricted to lower vertebrates. It has been known since the 1970s that the fingertips of children and adults regrow after distal amputation [16, 17]. Later, amputation level-dependent digit tip regeneration was also observed in mice [18, 19], which allowed the investigation of the cellular and molecular details of mammalian epimorphic regeneration, and to induce regeneration in non-regenerative amputation wounds [20, 21]. The terminal phalanx bone (P3) is the largest structure in the digit tip, and following distal amputation, a blastema forms by a combination of osteoprogenitor recruitment [22–24] and dedifferentiation of mesenchymal cells [10, 11, 24]. Investigations into the mechanisms of digit regeneration have revealed that non-regenerative proximal P3 amputation injuries have the cellular prerequisites necessary to elicit a modest regeneration response [25]. Recently, growth factors identified in P3 regeneration were found to induce bone and synovial joint regeneration of otherwise non-regenerative amputation injuries [21, 26]. In light of this new paradigm for inducing regeneration by activating endogenous regenerative potential, determining whether blastema-mediated regeneration is exhaustible in mammals is fundamental to understanding whether past injuries can render the amputation site refractory to a regeneration-inductive treatment, which may influence the utility of any future regenerative interventions. In this study, we repeatedly amputated digit tips in mice, and found that epimorphic regeneration ultimately fails after four rounds of repeated amputation. The results indicate that repeated amputations not only exhaust the local cell source to produce a functional blastema, but also induce systemic changes that are unfavorable for the epimorphic regeneration of previously uninjured tissues.

## Methods

### Animals and repeated digit tip amputations

Adult 8-week-old, female CD-1 mice were purchased from Texas Institute for Genomic Medicine (College Station, TX) and used for all experiments. For age-matched control experiments, the same mice were purchased and allowed to age until 6 months. All animal use and techniques were compliant with standard operating procedures and approved by the Texas A&M University Institutional Animal Care and Use Committee. Digit tip amputations of the 2nd, 3rd, and 4th hindlimb digits and µCT scans were performed on animals anesthetized with 2% isoflurane in oxygen as described previously [27].

**Fig. 1:**
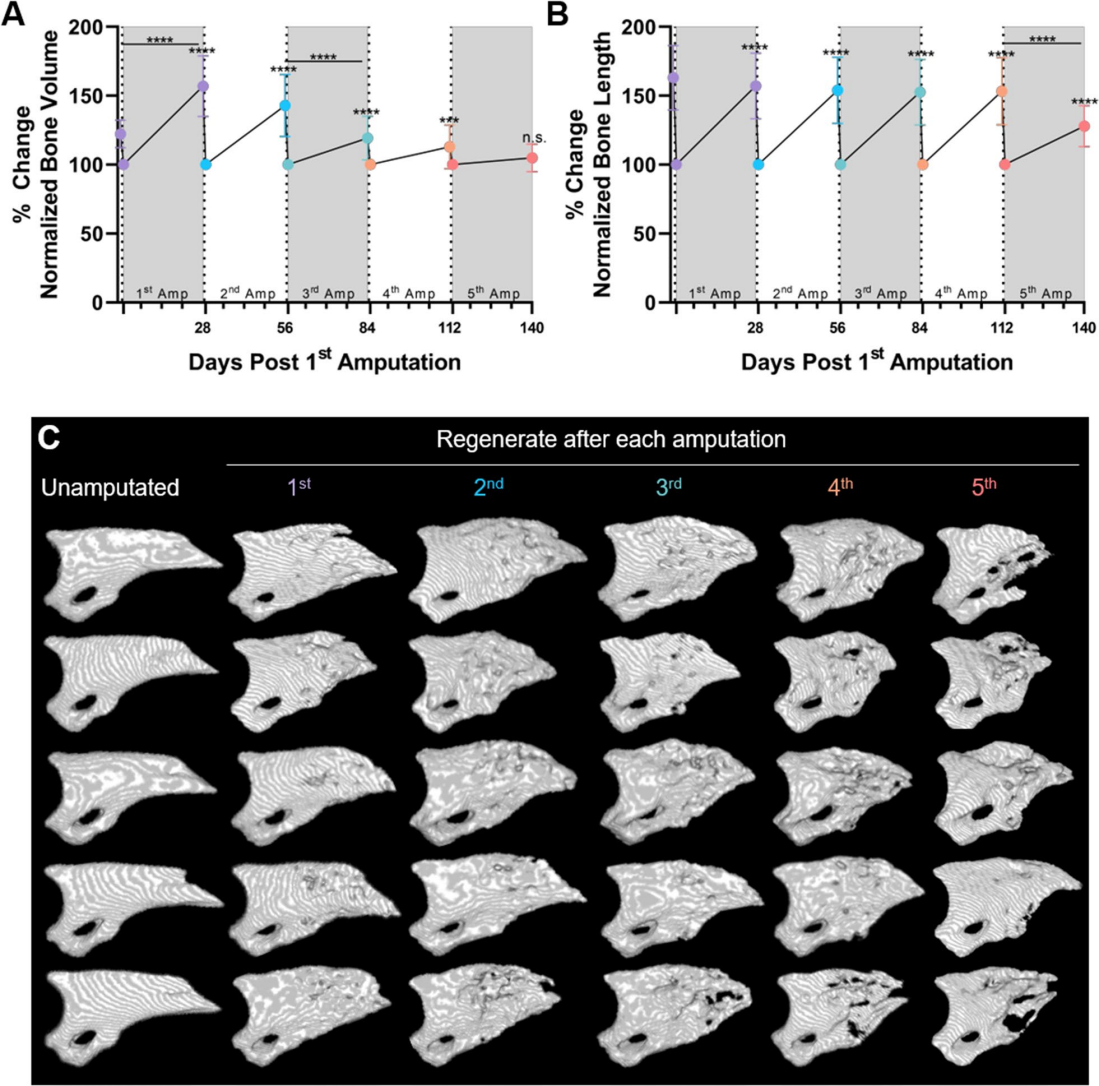
Repeated amputations inhibit digit tip regeneration. **A** Quantification of terminal phalanx (P3) bone volume and **B** P3 bone length normalized to the amputated bone volume (1 DPA) after each amputation. Asterisks indicate differences between amputated and regenerated digits for each regeneration cycle unless a bar is used to denote a specific comparison. **A**, **B** Black dashed lines indicate when digit tip amputation occurred. *n* = 40 digits; Differences were determined using a mixed-effects model with matching analysis test and a Tukey’s multiple comparisons test. Data are presented as mean ± SD; * = *p* < 0.05; *** = *p* < 0.001; **** = *p* < 0.0001; n.s. = not significant. **C** 3D µCT renderings of unamputated and regenerated P3 bones 28 days following amputation. A sample of 5 digits, longitudinally imaged during the experiment (same digit repeatedly imaged in each row) is presented to demonstrate variation

**Fig. 2:**
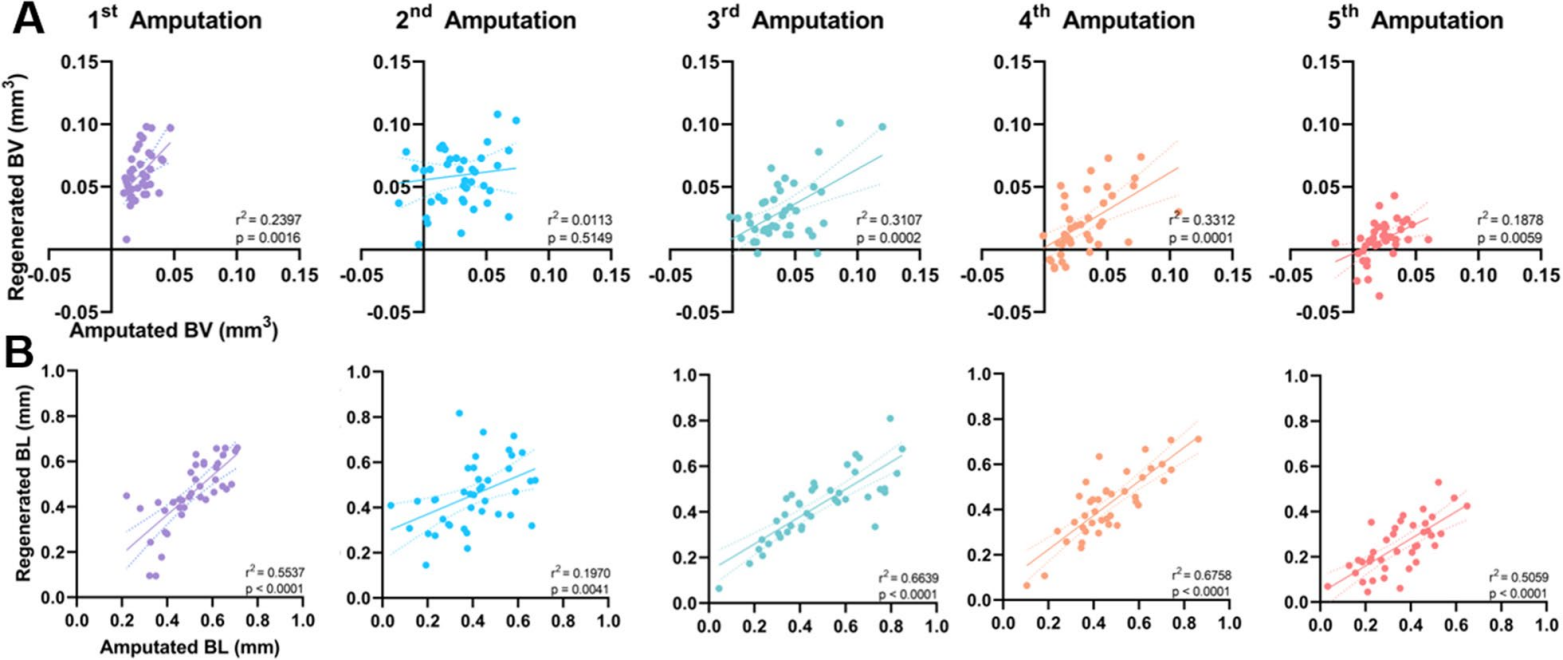
The correlation between the amount of amputated and regenerated bone is maintained. **A** Simple linear regression between the amount of amputated and regenerated terminal phalanx (P3) bone volume (BV) and **B** bone length (BL) after each amputation. **A**, **B** Line of best fit and 95% confidence intervals are presented as solid and dashed lines, respectively. *n* = 39–40 digits

**Fig. 3:**
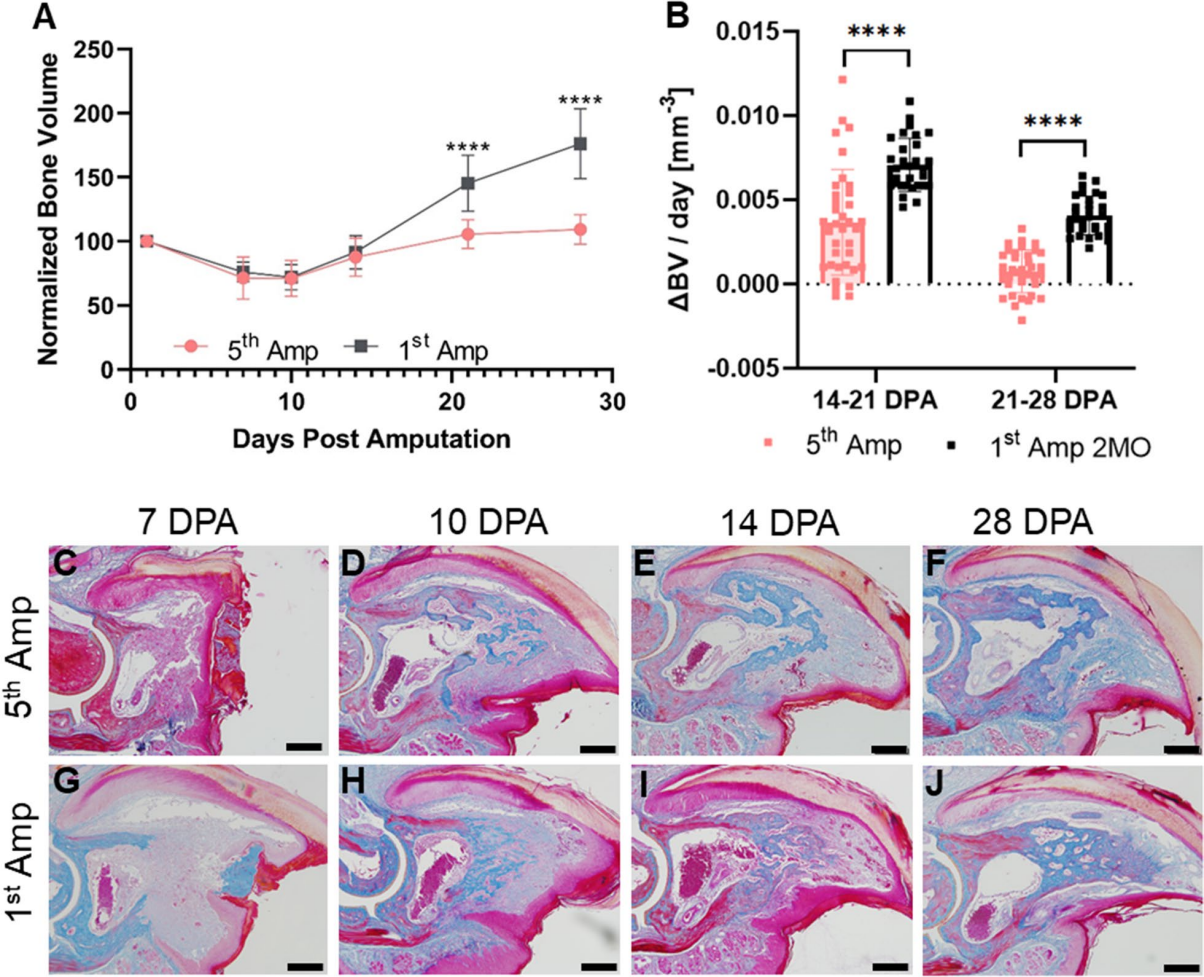
Repeated amputation stunts the anabolic phase of digit bone regeneration. **A** Quantification of normalized bone volume measurements for digits amputated 5 times (5th Amp; *n* = 32–52 digits) and control digits amputated for the first time (1st Amp 2MO; *n* = 18–26 digits). **B** Rates of bone volume changes calculated from (**A**). Negative values indicate bone degradation rate, positive values bone formation rate. **C**–**J** Mallory Trichrome-stained digit sections of 5th Amp digits (upper panel) and 1st Amp sections (lower panel) at wound closure and blastema initiation (**C**, **D**, **G**, **H**) and bone formation (**E**, **F**, **I**, **J**). Scale bar indicates 200 µm. Data are presented as mean ± SD and significant differences were determined using an unpaired *t* test. * = *p* < 0.05; ** = *p* < 0.01; *** = *p* < 0.001; **** = *p* < 0.0001

**Fig. 4:**
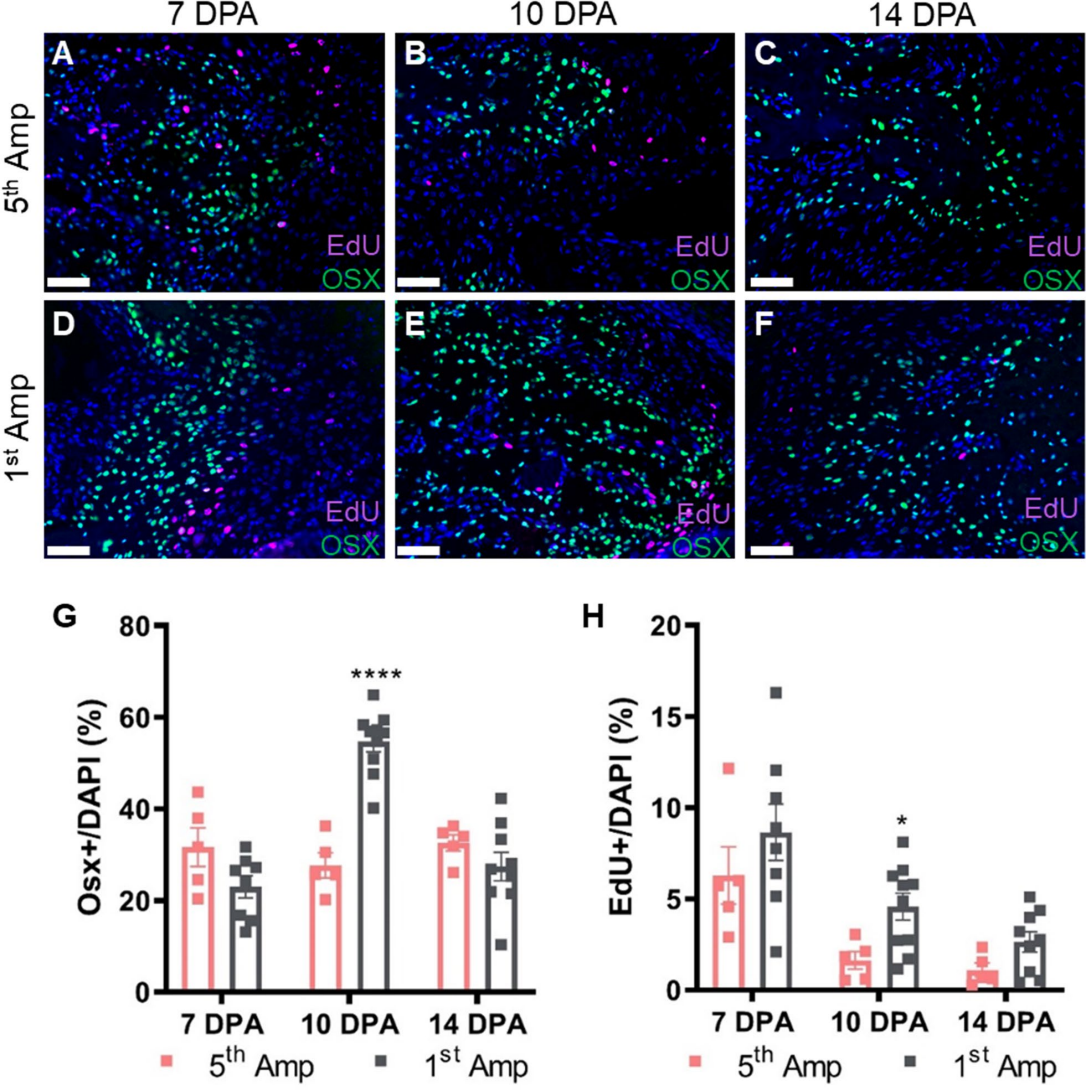
Repeated amputation inhibits blastema cell expansion and differentiation. **A**–**F** Representative images of 5th Amp (upper panel) and 1st Amp blastemas (lower panel) stained for osteoblasts (osterix, green) and proliferating cells (EdU, magenta) from 7 to 14 DPA. Sections were counterstained with DAPI (blue). **G**, **H** Immunohistochemical quantification of osteoblasts (Osx+) and proliferating cells (EdU+) normalized over DAPI in regenerating digits after the 5th amputation (*n* = 5 digits) and 1st amputation (*n* = 8–10 digits). Scale bar indicates 50 µm. Data are presented as mean ± SD and differences were determined using an unpaired *t* test. * = *p* < 0.05; ** = *p* < 0.01; *** = *p* < 0.001; **** = *p* < 0.0001

For repeat amputation experiments (Experiment 1; Figs. 1, 2), hindlimb digits were scanned using a μCT to obtain the unamputated bone volume and length measurements. After the scan was completed, digit tip amputations were performed on the 2nd and 4th digits of both hindlimbs (*n* = 4 digits/animal). The next day (1 day post-amputation), hindlimb digits were re-scanned, allowed to regenerate for 4 weeks (28 days post-amputation), re-scanned, and then re-amputated. This process was repeated for a total of 5 amputations. To assess the effect of repeated amputations on the individual phases of digit bone regeneration (Experiment 2; Figs. 3, 4), separate mouse cohorts were subjected to 4 amputation–regeneration cycles as above. During the final (5th) amputation–regeneration cycle, µCT scans were performed at 1, 7, 10, 14, 21 and 28 DPA, and digit tissues were collected at 7, 10, 14 and 28 DPA.

**Fig. 5:**
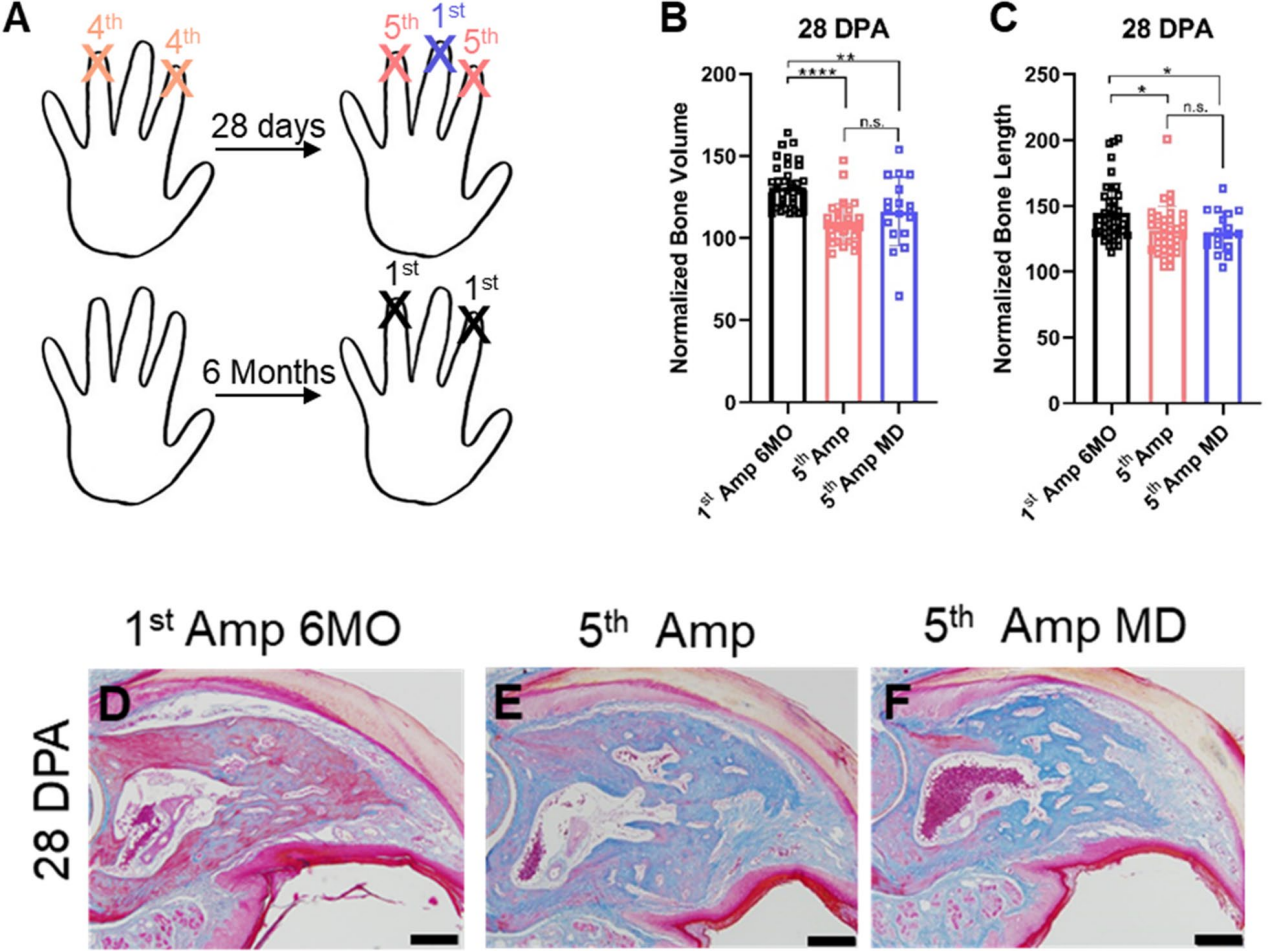
Attenuated regeneration of digits adjacent to repeatedly amputated digits. **A**–**C** Experimental outline of experiment 3 (**A**) (see “Methods” for description) and normalized bone volume (**B**) and length (**C**) of adjacent digits amputated for the first time (5th Amp MD; *n* = 18), compared to digits amputated for the 5th time (5th Amp, *n* = 36 digits), and digits of a separate mouse cohort of the same age amputated for the first time (1st Amp 6MO; *n* = 39 digits). **D**–**F** Mallory Trichrome-stained sections of age-matched control digits amputated for the first time (**C**), digits amputated for the 5th time (**D**), and adjacent middle digits amputated for the first time (**E**). Scale bar indicates 200 µm. Data are presented as mean ± SD and differences were determined using an unpaired *t* test. * = *p* < 0.05; ** = *p* < 0.01; *** = *p* < 0.001; **** = *p* < 0.0001

**Fig. 6:**
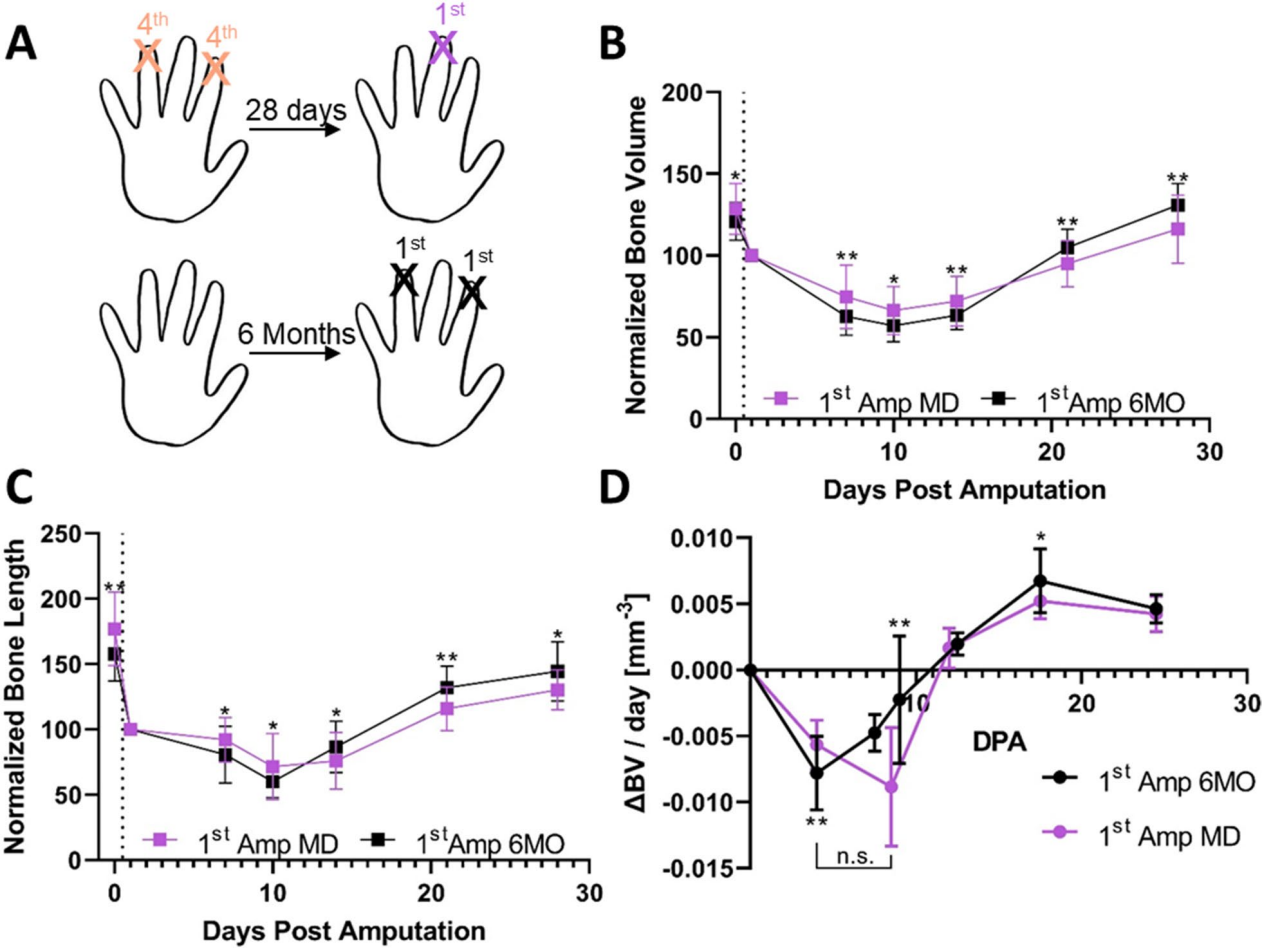
Repeated amputation has systemic effects on digit tip regeneration. **A** Experimental outline of experiment 4 (see “Methods” for description). **B**–**D** Quantification of normalized bone volume (**B**), bone length (**C**) and rate of volume change (**D**) for digits amputated 5 times (5th Amp; *n* = 32–52 digits) and middle digits amputated for the first time, where the 2nd and 4th hindlimb digits went through 4 amputation–regeneration cycles but were not amputated for a 5th time. (1st Amp MD; *n* = 20 digits). Data are presented as mean ± SD and differences were determined using an unpaired *t* test. * = *p* < 0.05; ** = *p* < 0.01; *** = *p* < 0.001; **** = *p* < 0.0001. Black dashed lines indicate when digit tip amputation occurred

For middle digit amputations (Experiment 3; Fig. 5), digits 2 and 4 of one mouse cohort were subjected to 4 amputation–regeneration cycles as above. At the time of the 5th amputation for the 2nd and 4th digits, the 3rd digit (middle digit) on each hindlimb (*n* = 2 digits/animal) was amputated for the first time, and µCT scans were performed at 1 and 28 DPA. For the final middle digit amputation experiment (Experiment 4; Fig. 6), digits 2 and 4 of one mouse cohort were subjected to 4 amputation–regeneration cycles as above. For the 5th amputation–regeneration cycle, only the 3rd digit (middle digit) on each hindlimb was amputated, while digits 2 and 4 were left intact. µCT scans were performed at 7, 10, 14, 21 and 28 DPA. Simultaneous regeneration of 1–6 digits (Additional file 1: Fig. 2): For determining if the number of regenerating digits at one time affected overall regeneration, 1, 2, 3, 4, or 6 digits were amputated per animal. The digit(s) amputated were the same digits for each animal: 1 amputation: L4, 2 amputations: L4/R4, 3 amputations: L4/L2/R4, 4 amputations: L4/L2/R2/R4, 6 amputations: L4/L3/L2/R2/R3/R4.

### Micro-computed tomography (μCT), image processing, and quantification of bone volume and bone length

Terminal phalanx (P3) bone volume and length were measured using a vivaCT 40 (SCANCO Medical) as previously described [27]. μCT files were saved as a DICOM image stack, and subsequently uploaded to ImageJ where we used the 3D viewer plugin to create 3D renderings of bones. Bone volume was measured using the volume fraction tool in the BoneJ plugin in ImageJ. Bone length was measured using the point tool also in ImageJ. Bone volume and length are either presented as absolute values (in mm^3^ and mm, respectively) or are normalized to each individual digit’s volume or length 1 day post-amputation to control for variation in digit size and amputation level. The amount of bone volume or length amputated was calculated with the formula 28 DPA^1st Amp^ – 1 DPA^2nd Amp^, 28 DPA^2nd Amp^ – 1 DPA^3rd Amp^, etc. The amount of bone volume or length regenerated was calculated with the formula 28 DPA^1st Amp^ – 1 DPA^1st Amp^, 28 DPA^2nd Amp^ – 1 DPA^2nd Amp^, etc. For bone degradation and bone formation rates, first derivatives, i.e., slopes, of individual curves were calculated using GraphPad Prism 9 (GraphPad Software).

#### Digit processing, immunohistochemistry, and EdU Injections

Digits were collected from mice and fixed in buffered zinc formalin (Anatech Ltd, Battle Creek, Michigan) for 24 h at room temperature. Digits were decalcified using Decalcifier I (Surgipath, Richmond, IL), a 10% formic acid solution, for 24 h. Decalcified digits were processed through a graded ethanol series, xylenes, and immersed in paraffin wax. Digits embedded in paraffin wax were serial sectioned at 4–5 μm thickness. Before immunohistological staining, slides were incubated at 60 °C for 45 min, followed by incubation at 37 °C for 2 h, with subsequent deparaffinization with xylenes, a graded ethanol series, and eventual submersion in water. Antigen retrieval was performed using heat retrieval performed in citrate buffer (Dako, Glostrup, Denmark). Slides were blocked using Protein Block (Dako) for 1 h at room temperature. Incubation with primary antibody was performed overnight at 4 °C; they were then washed in Tris buffered saline with Tween R20 solution (Sigma-Aldrich, St. Louis, MO) and incubated in secondary antibody for 1 h at room temperature. Slides were then incubated in a phosphate buffered saline (Sigma-Aldrich) with DAPI (Invitrogen) solution, dried, and mounted with Prolong Gold (Invitrogen, Waltham, MA). Samples were imaged using an Olympus BX61 microscope with a Hamamatsu ORCA-ER camera via the Slidebook software (Intelligent Imaging Innovations Inc.) EdU (5-ethynyl-2′-deoxyuridine; Sigma-Aldrich) was injected intraperitoneally (10μL/kg) 3 h prior to specimen collection. Primary antibodies used were rabbit anti-Osterix (1:400; Abcam, Cambridge, UK; #ab22552) with the secondary antibody goat anti-rabbit Alexa Fluor-488 or 568 (1:500; Invitrogen).

#### Quantification of EdU and Osterix

To quantify EdU-positive cells and osteoprogenitors (osterix-positive cells), digits were imaged at 200× magnification using a Olympus BX61 microscope with a Hamamatsu ORCA-ER camera. Using Slidebook software, a region of interest (ROI; 400 × 300 μm) was manually determined and automatically quantified. For both Osterix and EdU, the number of immunopositive cells was automatically determined within the same ROI and was normalized to the number of DAPI^+^ cells within the ROI. Three sections per slide were analyzed, averaged, and combined with other digits for statistical analysis which consisted of an unpaired *t* test using GraphPad Prism 9 software.

### Statistics

Statistical analysis was performed using GraphPad Prism 9 (GraphPad Software). Depending on the experiment (see Figure Legends for precise test), we either used a paired or unpaired *t* test (two-sided), one-way ANOVA with a post hoc Tukey’s multiple comparisons test, a mixed-effects model with matching analysis test and a post hoc Tukey’s multiple comparisons test, or simple linear regression.

## Results

### Mouse digit tip regeneration is exhaustible

To determine the extent to which mouse digits can continually regenerate after repeated amputations, the distal digit tips of the 2nd and 4th hindlimb digits were amputated on each hindlimb (*n* = 40 digits/10 mice). The first amputation was performed at 2 months of age, and digits were allowed to regenerate for 4 weeks before they were subjected to re-amputation at the same plane. Quantitation of amputated and regenerated digit bone volume and bone length was performed using in vivo micro-computed tomography (μCT) at 1 and 28 days post-amputation (DPA). The length of time between imaging post-amputation was purposefully selected to minimize any potential radiation-induced effects of frequent µCT [28]. To account for individual differences in amputation and digit size, regenerated bone volume measurements were normalized to the amputated bone volume after each sequential amputation.

As we have previously described [29], the bone volume of the regenerated digit (28 DPA) exceeds the volume of the nascent digit bone after the 1st amputation (Fig. 1). As such, the bone volume of regenerated digits exceeds the volume of unamputated digits by 35% (*p* < 0.0001), and of amputated digits by 57% (*p* < 0.0001) (Fig. 1A). As previously demonstrated [30], digits amputated and regenerated for the second time surpassed the amputation volume by 43% (*p* < 0.0001), which was equivalent to the excess volume after the first regeneration cycle (*p* = 0.2562) (Fig. 1A). Following the 3rd amputation, regenerates were on average 19% larger than the amputated digits (*p* < 0.0001), which is significantly less than after the 2nd amputation (24%, *p* < 0.0001) (Fig. 1A) and denotes a reduction of regenerative capacity. This diminishing regeneration response continues through the subsequent amputations, with a 13% of excess bone volume over the amputated volume (*p* = 0.003) after the 4th amputation, and a failure to surpass amputated bone volume after the 5th amputation (*p* = 0.1194) (Fig. 1A). Plotting the absolute bone volume data for each amputation–regeneration cycle confirmed that the bone volume progressively increases to the end of the second amputation–regeneration cycle, and then progressively decreases until no new bone growth is observed after the 5th amputation (Additional file 1: Fig. 1A). Together, these data demonstrate that there is a progressive loss of regenerative capacity after the 3rd amputation that culminates in complete exhaustion of regenerative capacity after the 5th amputation.

### Pattern formation and size regulation are resistant to repeated amputations

Despite the marked decrease in bone volume, we observed that the regenerated digits still assume a triangular shape with a tapered end (Fig. 1C), suggesting that pattern formation is relatively stable throughout serial amputation. To quantify this observation, the restoration of bone length was measured as a proxy for pattern formation. In accordance with previous studies [31], the regenerated bone length does not exceed the original bone length (Fig. 1B, Additional file 1: Fig. 1B). Complete bone length regeneration after each amputation was observed through the fourth regeneration cycle; the average regenerated bone length (1st: − 5.9%, 2nd: − 9.2%, 3rd: − 10.5%, 4th: − 9.8%) is not different (1st: *p* = 0.0704, 2nd: *p* = 0.6542, 3rd: *p* = 0.3009, 4th: *p* = 0.6626) compared with the length of the unamputated digits (Fig. 1B). Only after the 5th amputation is the average regenerated bone length significantly shorter compared to the regenerate after the 4th amputation (− 25.3%; *p* < 0.0001), and 35.1% shorter compared to the bone length prior to amputation (*p* < 0.0001) (Fig. 1B). These data demonstrate that in contrast to bone volume regeneration, which progressively declines with each amputation, bone length is maintained through four amputation–regeneration cycles and abruptly fails after the 5th amputation.

The extent of regeneration is proportional to the amputation level in salamander limbs, fish fins and mouse digit tips, suggesting an inherent positional memory that controls the size of the regenerate [32–34]. To determine whether size regulation is maintained after repeated amputations of the digit tip, the amount of regenerated bone was plotted against the amount of amputated bone. With the exception of the second amputation–regeneration cycle, both amputated bone volume and bone length were predictive of the amount regenerated (Fig. 2A, B). For bone volume, after all except the 2nd amputation where there is no relationship (*p* = 0.5149), amputated volume explains between 19 and 33% of the variability in regenerated bone volume (Fig. 2A). For bone length however, after all 5 amputations, amputated length explains between 20 and 68% of the regenerated bone length and is more strongly correlated with the amount regenerated than bone volume after every amputation (Fig. 2B). These data indicate that repeated amputation does not inhibit the mechanisms of pattern formation and size regulation active in the regenerating mouse digit tip.

### Repeated amputation attenuates bone formation

Digit tip regeneration proceeds through a series of coordinated events which can be grouped into two overlapping phases: a catabolic and an anabolic phase [29, 31]. After amputation, the P3 bone is degraded by bone-resorbing osteoclasts, leading to a significant reduction in both bone volume and bone length. This initial catabolic phase is followed by an extensive period of osteoblast-mediated bone formation that leads to the reestablishment of bone volume and bone length by 28 DPA [31]. To determine whether regenerative failure is associated with excessive bone destruction or the inhibition of bone formation, or a combination of both, digit tips were amputated every 28 days and changes in bone volume and length regeneration quantified using in vivo μCT during the 5th amputation–regeneration cycle (*n* = 52 digits). Compared to digits amputated for the first time, no excessive osteoclast-mediated bone degradation was observed (1–10 DPA) in digits amputated 5 times (5th Amp) (10 DPA: *p* = 0.7151) (Fig. 3A). However, during the anabolic bone formation phase, 5th Amp digits exhibit a significant inability to regenerate new bone, and at 21 and 28 DPA, are 39.7% (*p* < 0.0001) and 67% (*p* < 0.0001), respectively, smaller than digits regenerating for the first time (Fig. 3A). The attenuation of bone regeneration was reflected in a slower bone formation rate and a premature termination of bone formation activity in 5th Amp digits (Fig. 3B). Digit tissue histology suggests that the stunted bone growth is associated with a disorganized structure of the connective tissue reminiscent of fibrotic tissue [25] (Fig. 3F), while epidermal wound closure and blastema initiation appeared to be comparable (Fig. 3C–J), which is consistent with the synchronous transition from the catabolic to the anabolic phase in 5th Amp and control digits (Fig. 3A). To investigate the cellular underpinnings of these observations, digits were harvested during different stages of blastema formation (7, 10, and 14 DPA) and the number of osteoblasts and the cell proliferation index determined. Both osteoblast numbers and proliferation index were comparable at blastema recruitment at 7 DPA (Fig. 4A, D, G, H). At 10 DPA, digits amputated 5 times had 65.7% (*p* < 0.0001) fewer osteoblasts and 94.8% (*p* = 0.0204) fewer proliferating cells compared to digits amputated for the first time (Fig. 4B, E, G, H). Taken together, these results suggest that the low rate and premature termination of bone formation resulting in regenerative failure after repeated amputations are rooted in an osteoblast differentiation defect and/or proliferative exhaustion of the blastema cells. Since the blastema proliferation index and the number of osteoblasts early in the regeneration response are not affected by repeated amputation, recruitment of osteoprogenitor cells to form the blastema appears to be normal.

### Repeated amputations induce systemic attenuation of regeneration

The digit tip regeneration studies herein were performed on digits 2 and 4 of both hind paws, leaving the middle digit (digit 3; MD) undisturbed [31]. In an attempt to reduce the required mouse numbers and the variability between experimental and control digit cohorts and based on the paradigm that epimorphic appendage regeneration is controlled locally [15, 35], we reasoned that previously unamputated middle digits would be a suitable internal control for repeated amputation studies. Therefore, for the fifth and last amputation–regeneration cycle, digit 3 of both hindpaws (R3 and L3, amputated for the first time; 5th Amp MD) was amputated in addition to digits 2 and 4 (R4, R2, L4, L2; amputated for the 5th time; 5th Amp) (Fig. 5A) (for details see description of Experiment 3 in “Methods”), with the hypothesis that bone volume and length would be fully regenerated at the end of the cycle. Surprisingly, the regenerated bone volume (*p* = 0.2204) and length (*p* = 0.9817) were similar between the 5th Amp and 5th Amp MD digits, and smaller than age-matched digits that had undergone only one amputation–regeneration cycle (1st Amp 6MO) (Fig. 5B–F). Thus, digits adjacent to repeatedly amputated digits do not fully regenerate. After excluding the possibility that the number of digits regenerating at the same time affects regeneration (Additional file 1: Fig. 2), we reasoned that the diminished regeneration of middle digits is mediated by systemic alterations induced by repeated amputations. To test this hypothesis, hindlimb digits 2 and 4 were subjected to four amputation–regeneration cycles as described above. After completion of the 4th cycle, only the middle digits were amputated (1st Amp MD), with changes in bone volume and length monitored by in vivo µCT up to 28 DPA and compared to a separate age-matched cohort (1st Amp 6MO) (Fig. 6A) (Experiment 4 in Methods). The middle digits exhibited attenuated regeneration compared to age-matched control digits (Fig. 6B, C). This attenuation was associated with slower bone resorption and bone formation, reminiscent of the effects of aging on digit regeneration [36] (Fig. 6D). Taken together, these data suggest that a total of 16 amputation–regeneration cycles (4 digits amputated 4 times) induce significant systemic changes that disrupt the regenerative capacity of adjacent, previously uninjured digits.

## Discussion

The blastema that forms following amputation of the mouse digit tip is composed of a variety of cell types, the majority of which are multipotent mesenchymal cells derived from the endosteal/marrow cavity and the periosteum [10, 22–24, 31]. If a regeneration cycle is to be repeatable, the nascent periosteal and endosteal niches must be repopulated with new progenitors that are still able to proliferate sufficiently and differentiate in time, such that faithful regeneration occurs. These fundamental physiologic requirements are fulfilled in planaria, newts and zebrafish, where repeated amputation injuries do not lead to regenerative failure [12, 13, 37]. On the other hand, axolotl limbs undergo a finite number of regeneration cycles, and it has been suggested that this is either due to a decline in progenitor cells or a stunted limb growth phase [15, 38]. In order to answer the question of whether mammals completely replenish the blastema cell source, we repeatedly amputated digit tips in female mice and found that epimorphic regeneration ultimately fails, suggesting that repeated regeneration cycles exhaust progenitor cell sources for the blastema. Notably, our data indicate that blastema cell recruitment is not affected, because blastema proliferation and osteoprogenitor numbers are normal at an early regeneration stage. Therefore, in the regenerating mouse digit tip, repeated amputation does not lead to a decline in progenitor cells, but a stunted growth phase.

As measured by bone volume, the mouse digit is only capable of complete regeneration for two regeneration cycles. However, regenerate bone length is re-established for four regeneration cycles and is directly proportional to the length amputated in the respective cycle. Moreover, the gross shape of the regenerated digit is maintained even until the end of the fifth amputation–regeneration cycle. This indicates that positional memory necessary for de novo skeletal morphogenesis resists multiple injuries and regeneration cycles. Consistent with our findings, positional information is stable in the axolotl and zebrafish [32, 39], as well as in mammalian cells: Fibroblasts maintain their positional identity when cultivated in vitro [40], and tibial-derived mesenchymal skeletal progenitor cells maintain their appendicular skeletal identity when transplanted into a mandibular defect [41]. This stability of positional information may explain the prioritization for regenerating digit length, which only fails abruptly after the fourth amputation–regeneration cycle when the propagation of osteoprogenitor cells fails. These studies are consistent with and support the distinction between pattern-forming cells and pattern-following cells, in this case osteoprogenitors, described in other models of epimorphic regeneration [42].

In vertebrates, epimorphic regeneration is controlled and executed by cells adjacent to the amputation site and is considered to be unperturbed by systemic factors [15, 35]. In the mouse digit model, this paradigm is supported by findings that circulating cells do not contribute to the final regenerate [23]. We therefore reasoned that naïve digits adjacent to previously amputated digits would serve as an ideal internal control for repeated amputation experiments. Surprisingly, digits that were amputated for the first time, but adjacent to digits that have undergone multiple regeneration cycles, were also significantly impaired in regenerative ability. Subsequently, we demonstrated that the impaired regenerative response was not a consequence of age or the number of simultaneously regenerating digits. The most parsimonious explanation for these findings could be that, at least in the context of repeated amputations, there are as yet unidentified systemic components regulating mammalian digit tip regeneration. While local effectors of epimorphic regeneration have been the primary focus in the last decades, both cellular and humoral systemic mediators of epimorphic regeneration have been explored [43, 44]. With the exception of deer antler regeneration [45], no such studies exist for mammalian epimorphic regeneration until now. As mentioned above, circulating cells do not contribute to the final regenerate [23]. This, however, does not exclude the possibility that circulating cells transiently contribute to and modify the course of regeneration; in fact, previous studies have shown that circulating monocytes, macrophages, neutrophils and T cells are recruited to the regenerating digit in the early stages [11, 46]. Given that four amputation–regeneration cycles of one month each subject a mouse to repetitive inflammation and healing cycles for roughly 10% of its life, and that the changes in digit bone regeneration are reminiscent of changes associated with chronological aging [36], it is reasonable to presume that repeated amputations induce lasting changes in the cellular and humoral immune response akin to inflammaging [47] and consequently affect regeneration of previously uninjured tissues.

A remaining unanswered question is what distinguishes planaria, zebrafish and newts, which seemingly regenerate indefinitely [12, 13, 37], from axolotl limbs and mouse digits with a limited capacity for epimorphic regeneration. One possible distinction is the source of progenitor cells. The source of planarian regeneration is ubiquitous, pluripotent stem cells (neoblasts) that do not require a localized niche and are highly plastic [48]. Newt lenses are regenerated by dedifferentiated iris cells [49], and zebrafish fins utilize both committed and dedifferentiated osteoblast populations [50]. In contrast, blastema cells for axolotl limb and mouse digit regeneration are recruited from local sources and are lineage restricted. Axolotl muscle regenerates by recruiting local satellite stem cells [51], and mouse digit blastema cells are derived from local mesenchymal cells [10, 11, 23]. These data support the idea that the predominant utilization of lineage-restricted progenitor cells, as opposed to a predominant use of pluripotent or dedifferentiated cells, may be the distinguishing feature between species that are limited in their regenerative capacity and species with apparent indefinite regenerative capacity.

## Conclusion

To conclude, the data presented herein demonstrate that mammalian epimorphic regeneration is exhaustible. The regenerative failure of repeatedly amputated digits is associated with a premature termination of blastema cell proliferation, not a failure of blastema cell recruitment. These results indicate that repeated amputations exhaust the local progenitor cell reservoirs for blastema formation and provide the first evidence for an important systemic component to mammalian epimorphic regeneration. Ongoing exploration will be needed to determine the relative contribution of local and systemic factors, in order to enhance regeneration in non-regenerative amputation wounds in higher mammals, such as humans.

## Supplementary Information

**Additional file 1. Supplemental Figure 1:** Digit tip regeneration is inhibited by repeated amputations (**A**) Terminal phalanx (P3) bone volume and (**B**) length after each amputation. Black dashed lines indicate when digit tip amputation occurred. n = 40 digits; Differences were determined using a mixed-effects with matching analysis test and a Tukey’s multiple comparisons test. Data presented as mean ± SD; * = *P*<0.05; *** = *P*<0.001; **** = *P*<0.0001; n.s. = not significant. **Supplemental Figure 2**: Number of regenerating digits does not affect net regeneration. (**A**) Quantification of normalized bone volume at 28 days post amputation (DPA) where either 1 (n = 10), 2 (n = 10), 3 (n = 15), 4 (n = 20), or 6 (n = 30) digits were regenerating at once. Data presented as mean ± SD

## Data Availability

All data are available in the main text or the supplementary materials.
