# Supplementary material for: Epimorphic regeneration of the mouse digit tip is finite
Source: Stem Cell Res Ther. 2022 Feb 7;13:62. doi: 10.1186/s13287-022-02741-2 (PMC8822779; doi:10.1186/s13287-022-02741-2)

**SUPPLEMENTAL FIGURES**

**Supplemental Figure 1: Digit tip regeneration is inhibited by repeated amputations**

**(A)** Terminal phalanx (P3) bone volume and **(B)** length after each amputation. Black dashed lines indicate when digit tip amputation occurred. n = 40 digits; Differences were determined using a mixed-effects with matching analysis test and a Tukey’s multiple comparisons test. Data presented as mean ± SD; * = P<0.05; *** = P<0.001; **** = P<0.0001; n.s. = not significant.


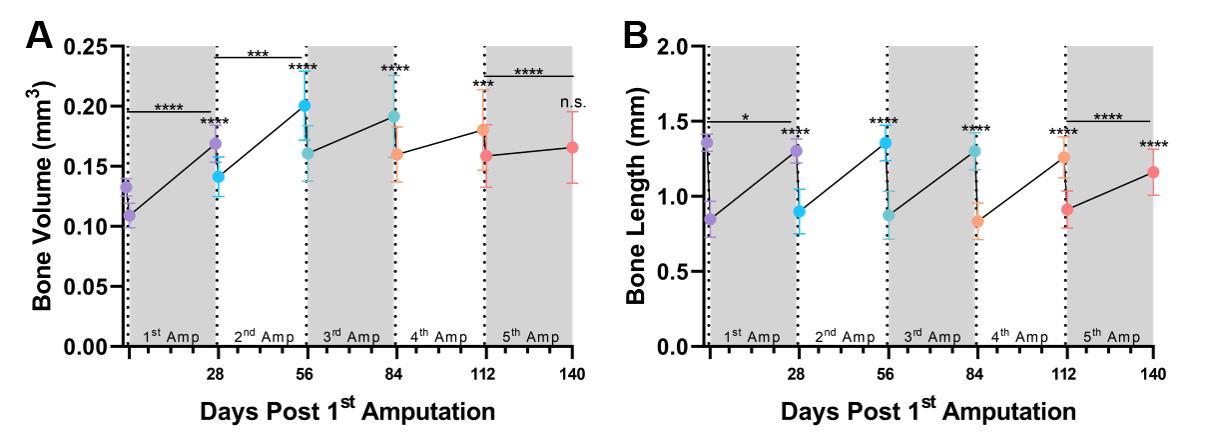


**Supplemental Figure 2: Number of regenerating digits does not affect net regeneration.**

(A) Quantification of normalized bone volume at 28 days post amputation (DPA) where either 1 (n = 10), 2 (n = 10), 3 (n = 15), 4 (n = 20), or 6 (n = 30) digits were regenerating at once. Data presented as mean ± SD.


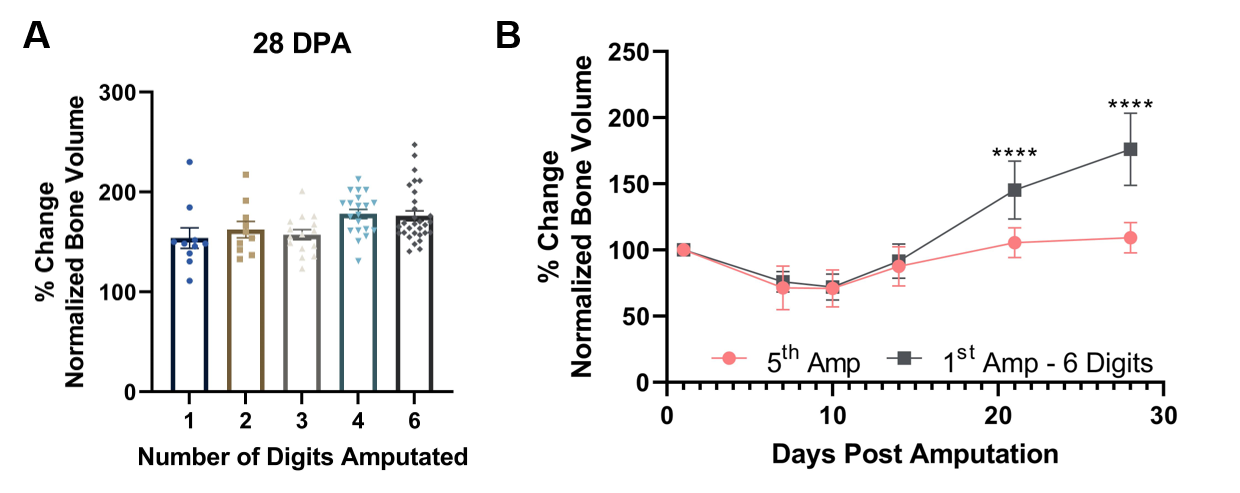

Supplement: Supplementary file 1 — Additional file 1. Supplemental Figure 1: Digit tip regeneration is inhibited by repeated amputations (A) Terminal phalanx (P3) bone volume and (B) length after each amputation. Black dashed lines indicate when digit tip amputation occurred. n = 40 digits; Differences were determined using a mixed-effects with matching analysis test and a Tukey’s multiple comparisons test. Data presented as mean ± SD; * = P<0.05; *** = P<0.001; **** = P<0.0001; n.s. = not significant. Supplemental Figure 2: Number of regenerating digits does not affect net regeneration. (A) Quantification of normalized bone volume at 28 days post amputation (DPA) where either 1 (n = 10), 2 (n = 10), 3 (n = 15), 4 (n = 20), or 6 (n = 30) digits were regenerating at once. Data presented as mean ± SD [file 13287_2022_2741_MOESM1_ESM.docx]
